# Mobile Digital Health Intervention to Promote Nutrition and Physical Activity Behaviors Among Long-term Unemployed in Rural Areas: Protocol for a Randomized Controlled Trial

**DOI:** 10.2196/40321

**Published:** 2022-11-14

**Authors:** Iris Weishaupt, Jennifer Mages-Torluoglu, Christophe Kunze, Christian Weidmann, Kirsten Steinhausen, Anja Christina Bailer

**Affiliations:** 1 Institute for Applied Health Promotion and Exercise Medicine (IfAG) Faculty of Health, Safety, Society Furtwangen University Furtwangen Germany; 2 Institute of Sociology Faculty of Educational Sciences University of Education Freiburg Freiburg Germany; 3 University of Applied Sciences Fulda Fulda Germany; 4 Care & Technology Lab (IMTT) Faculty of Health, Safety, Society Furtwangen University Furtwangen Germany

**Keywords:** digital health intervention, behavior changes, nutrition, physical activity, long-term unemployment, rural areas, Germany, mobile phone

## Abstract

**Background:**

Long-term unemployed have poor nutritional and physical activity statuses, and, therefore, special health promotion needs. Particularly in rural areas, however, they often do not have access to health promotion service. Thus, new promising strategies to improve the health of long-term unemployed are needed. Hence, a digital health intervention to promote nutritional and physical health behaviors was conceived, and the effectiveness of the intervention in combination with face-to-face sessions will be evaluated in a randomized controlled trial.

**Objective:**

The aim of this study is to elucidate the effectiveness of a mobile digital health intervention to promote the nutritional and physical activity behaviors of long-term unemployed in the rural areas of Germany.

**Methods:**

The 9-week intervention aims to promote nutritional or physical activity behavior by improving drinking habits, increasing the consumption of fruits, vegetables, and whole grains, increasing daily step count, strengthening muscles, and improving endurance. The intervention design is based on the transtheoretical model and is implemented in a mobile app using the MobileCoach open-source platform. The effectiveness of the intervention will be elucidated by a 9-week, 2-armed, parallel-designed trial. Therefore, long-term unemployed will be recruited by employees of the German social sector institutions and randomized either to receive information brochures; the digital intervention in the form of a mobile app; and 3 face-to-face sessions regarding technical support, healthy eating, and physical activity (n=100) or to receive a control treatment consisting of solely the hand over of information brochures (n=100). The effectiveness of the intervention will be assessed using questionnaires at baseline, after 9 weeks in face-to-face appointments, and after a 3-month follow-up period by postal contact. The use of the mobile app will be monitored, and qualitative interviews or focus groups with the participants will be conducted. Incentives of €50 (US $49.7) will be paid to the participants and are tied to the completion of the questionnaires and not to the use of the mobile app or progress in the intervention.

**Results:**

The effectiveness of the intervention in promoting the nutritional and physical activity behaviors of long-term unemployed participants will be elucidated. The adherence of the participants to and the acceptance and usability of the mobile device app will be evaluated. Recruitment started in March 2022, and the final publication of the results is expected in the first half of 2023.

**Conclusions:**

Positive health-related changes made by the intervention would display the potency of digital health interventions to promote nutritional and physical activity behaviors among long-term unemployed in the rural areas of Germany, which would also contribute to an improved health status of the German population in general.

**Trial Registration:**

German Clinical Trials Register DRKS00024805; https://www.drks.de/DRKS00024805

**International Registered Report Identifier (IRRID):**

PRR1-10.2196/40321

## Introduction

### Background

As of 2020, approximately 7.2% of the total working population was unemployed around the globe [1]. This condition was exacerbated by the COVID-19 pandemic. In Germany, the rate of unemployment rose from 5.3% in prepandemic January 2020 to 6.3% in January 2021 [2,3]. Although the unemployment rate dropped again to 5.4% in January 2022, approximately 2.5 million people of the German population were unemployed, of which 40.2% were without work for >12 months [4]. There is striking evidence that unemployment is associated with poor health outcomes (reviewed in the study by Jin et al [5]). In 2011, Roelfs et al [6] meta-analyzed that all-cause mortality was 63% higher in the unemployed population than in the working population. In addition to increases in mental health diseases, such as depression, anxiety disorders [7], psychoses [8], and substance abuse [9], physical diseases also occur more often in the unemployed than in the employed [10]. These include cancer [11] and cardiovascular events [12,13]. deBoer et al [11] compared participants who had cancer in recent years with cancer-free control participants and found statistically significant higher unemployment rates in the former, particularly in those with cancers of the gastrointestinal system (relative risk of 1.41). Moreover, Gallo et al [13] showed that the unemployed had 2.4- and 2.5-fold higher risks of stroke and myocardial infarction (MI), respectively. In this regard, the risk of MI increases with increasing length of unemployment. While the relative risk of MI was 1.49 times higher in participants who were unemployed for up to 8 months than in the employed participants, it was 3.08 times higher in those who were unemployed for >16 months [14]. Gastrointestinal cancer and cardiovascular events are also known to be influenced by poor nutrition (reviewed in the study by Wei et al [15]) and physical activity behavior (reviewed in the study by Lacombe et al [16]), which are generally common among the unemployed [17,18].

Although there is no difference in health-related behavior between long-term unemployed persons in urban areas and those in rural areas, the latter have more difficult access to health promotions from, for example, primary care physicians or offers from health insurances because of a lack of financial resources, poorly developed public transportation infrastructure, and thus lower mobility [19]. This shows that strategies to reach the long-term unemployed in rural areas are especially in need. Therefore, interventions designed in a digital format might be promising strategies to promote the health of the long-term unemployed in rural areas.

In general, digital health interventions (DHIs), particularly those on mobile devices, have been shown to be effective tools for inducing health-related behavior changes [20-23]. Moreover, conversational agents (CAs), computer programs that simulate conversations, are being increasingly used as DHIs [24], including behavior change apps to promote healthy eating [25] or physical activity [26]. However, DHIs require access to technology and sufficient knowledge to use it (digital literacy). Social health inequalities may contribute to a digital gap, as next to older age and being male, lower level of education and lower annual income are associated with a lower likelihood of owning a smartphone [27]. By contrast, Rhoades et al [28] showed that more than half of the homeless population owns smartphones and uses the internet daily. Moreover, Reinwand et al [29] reported that unemployed persons in a randomized controlled study used the intervention more frequently than employed persons, probably because it was time consuming and they had more time to use it.

### Objective

The aim of this study is to elucidate whether a DHI, conceived for use on mobile devices, can improve the nutritional and physical activity behaviors of long-term unemployed in the rural areas of Germany. Therefore, a customized 9-week intervention is conceptualized and implemented in a mobile app and will be tested in a randomized controlled trial (RCT). The effectiveness of the intervention will be assessed by questionnaire assessment.

## Methods

### Intervention Contents and Mobile App

The intervention design was planned in accordance with the intervention mapping approach [30]. The intervention content and the mobile app were designed through a user-centered approach including the needs assessment [31], a participatory design workshop with long-term unemployed (N=7), and a pretest for formative evaluation.

The intervention content is based on the transtheoretical model (TTM) for health behavior change by Prochaska et al [32,33]. The 9 weeks of the conceptualized intervention are adapted to the phases of the TTM as follows: week 1, “precontemplation”; week 2, “contemplation”; week 3, “preparation”; weeks 4 to 7, “action”; and weeks 8 to 9, “maintenance.” Before the intervention, the participants were assigned to week 1 (“precontemplation”), week 2 (“contemplation”), or week 3 (“preparation”) depending on their initially reported nutritional or physical activity behavior. The initially reported behavior was assessed by answering the following questions: “Do you regularly eat a balanced diet for example, foods such as fresh fruits and vegetables several times a week, or whole grains as well as dairy products sometimes and less sausage and meat?” or “Do you exercise regularly, for example, walking, biking, swimming, or going to the grocery store, that is, for at least 30 minutes each, at least 5 days per week?” If the questions were answered with “no,” the participant will receive the following selections: (a) “...and I do not think about eating a more balanced diet/exercising more,” (b) “...but I do think about eating a more balanced diet/exercising more,” or (c) “...but I will start eating a more balanced diet/exercising more.” The participant will (1) start in week 1 if the initial question is answered with “no” and then (a) is selected, (2) start in week 2 if the initial question is answered with “no” and then (b) is selected, or (3) start in week 3 if the initial question is answered with “yes” or with “no” and then (c) is selected. Therefore, the actual intervention duration varies between 7 and 9 weeks. The TTM stage is measured only once at the beginning of the intervention. However, the participants reach the next TTM stage only when they proceed to participate in the intervention. The intervention will be adaptive, and the participants must initially decide whether they want to promote their nutritional or physical behavior. Furthermore, in week 3 (“preparation”), they can choose to pursue only one of the following aims: (n1) change drinking habits, (n2) eat more fruits and vegetables, (n3) eat more whole grain products, (pa1) increase step count, (pa2) strengthen muscles, or (pa3) improve endurance, depending on whether they initially chose to promote nutritional (n1-3) or physical activity (pa1-3) behavior change. After the completion of the chosen aim, they will have the opportunity to select a new one. The intervention contents are designed in accordance with official national and international nutritional [34,35] and physical activity [36,37] recommendations. Detailed information about the intervention contents is provided in Tables 1 and 2. The language of the intervention is German.

**Table 1:** Intervention contents to promote nutritional behavior.

Phase of the TTM^a^ (week)	Pursued goals	Intervention content	Behavior change techniques according to Michie et al [38]
Precontemplation (1)	Recognize the benefits of healthy eating and the risks of unhealthy eating and perceive conducive environmental conditions that facilitate the change of problem behavior (unhealthy eating)	(1) Benefits of healthy eating and risk of unhealthy eating, (2) information about the food pyramid, (3) information about serving size and food frequencies, (4) information about macronutrients, and (5) information about micronutrients	Self-monitoring of behavior, information about health consequences, salience of consequence, prompts/cues, and pros and cons
Contemplation (2)	Recognize the added value of healthy eating for one’s own health and well-being and recognize the positive and negative consequences of current and target behavior for oneself and the environment	(1) Effects of healthy eating on digestion and well-being, (2) explanation on how to understand and use the Nutri-Score, (3) benefits of fresh foods, (4) information about food waste, and (5) information on various health parameters	Feedback on behavior, self-monitoring of behavior, self-monitoring of the outcomes of behavior, information about health consequences, salience of consequences, information about social and environmental consequences, demonstration of the behavior, pros and cons, and imaginary reward
Preparation (3)	Learning to set and pursue your own goals: (1) change drinking habits, (2) eat more fruits and vegetables, and (3) eat more whole grain products	(1) Self-reflection/self-image in relation to nutrition and nutrition habits, (2) committing to 1 out of 3 goals, (3) dealing with the weaker self, (4) building social relationships, and (5) pros and cons of the selected goal	Problem solving, action planning, discrepancy between current behavior and goal, self-monitoring of outcome(s) of behavior, feedback on the outcome(s) of behavior, social support (unspecified), verbal persuasion about capability, and pros and cons
Action (4-7)	Goal is pursued and implemented	(1) Overview, task, and information about weekly themes, recipes, and suggestions, (2) tips on planning purchasing, (3) tips to increase healthy nutrition in everyday life or leisure time, (4) motivation (push messages, positive feedback, and encouragement of participants’ abilities), (5) information about various nutrition themes, and (6) daily and weekly task checks	Goal setting (behavior), problem solving, feedback and monitoring, feedback on behavior, self-monitoring of behavior, comparison of behavior, demonstration of the behavior, repetition and substitution, practice/rehearsal, behavior substitution, generalization of target behavior, graded tasks, reduce negative emotions, self-belief, and verbal persuasion about capability
Maintenance (8-9)	Consolidation of the goal and identification of counterstrategies	(1) (Self-) Reward, (2) habit-building tips, (3) role models implementing healthy nutritional behavior, (4) successfully identifying and overcoming barriers, and (5) motivation (push messages, positive feedback, and encouragement of participants’ abilities)	Self-monitoring of behavior, instruction on how to perform the behavior, demonstration of the behavior, habit formation, nonspecific reward, self-reward, reduce negative emotions, and focus on past success

^a^TTM: transtheoretical model [32,33].

**Table 2:** Intervention contents to promote physical activity behavior.

Phase of the TTM^a^ (week)	Pursued goals	Intervention content	Behavior change techniques according to Michie et al [38]
Precontemplation (1)	Recognize the benefits of physical activity and the risks of physical inactivity and perceive conducive conditions that facilitate the change of problem behavior (physical inactivity)	(1) Benefits of physical activity for different parts of the body, (2) consequences of physical inactivity, (3) opportunities for physical activity in everyday life and sport, (4) World Health Organization recommendations, (5) highlighting the positive effects of everyday physical activity, (6) invitations to try physical activity, (7) reflection on everyday physical activity and sporting activities in youth, in old age, and today, and (8) highlighting resources in the own environment	Self-monitoring of behavior, instruction on how to perform the behavior, information about health consequences, salience of consequence, and prompts/cues
Contemplation (2)	Recognize the added value of physical activity for one’s own health and well-being, perceive the current state of physical activity, and recognize the positive and negative consequences of current and target behavior for oneself, the environment, and one’s surroundings	(1) Effects of physical activity on well-being and environment, (2) information on various health parameters, (3) perception of well-being and feelings through self-tests, tasks, and challenges, (4) possibility to do own calculations (eg, pulse measurement), (5) perceive body signals/get to know stress limits, (6) stimulating exchange with friends/acquaintances, and (7) independent reflection on the consequences of lack of physical activity in one’s own environment, now and in the past	Self-monitoring of outcome(s) of behavior, information about health consequences, salience of consequences, information about social and environmental consequences, monitoring of emotional outcomes, and reduce negative emotions
Preparation (3)	Learning to set and pursue your own goals: (1) increase step count, (2) strengthen muscles, or (3) improve endurance	(1) Self-reflection/self-image in relation to physical activity, (2) analyze strengths and weaknesses, (3) time management, (4) motivation, (5) build social relationships, (6) relaxation exercises, (7) behavioral observation and help to classify average activity and feedback, and (8) committing to 1 out of 3 goals	Problem solving, action planning, discrepancy between current behavior and goal, self-monitoring of outcome(s) of behavior, feedback on outcome(s) of behavior, information about antecedents, social support (unspecified), and verbal persuasion about capability
Action (4-7)	Goal is pursued and implemented	(1) Strategies on how to set and achieve a goal, (2) weekly and daily goals, (3) tips on different ways to increase physical activity in everyday life/leisure time, (4) motivation (push messages, positive feedback, and encouragement of participants’ abilities), and (5) training plans	Goal setting (behavior), problem solving, feedback and monitoring, feedback on behavior, self-monitoring of behavior, comparison of behavior, demonstration of the behavior, repetition and substitution, behavioral practice/rehearsal, behavior substitution, generalization of target behavior, graded tasks, reduce negative emotions, self-belief, and verbal persuasion about capability
Maintenance (8-9)	Consolidation of the goal and identification of counterstrategies	(1) (Self-) Reward, (2) habit-building tips, (3) role models implementing physical active rather than inactive behavioral alternatives, (4) successfully identifying and overcoming barriers, and (5) motivation (push messages, positive feedback, and encouragement of participants’ abilities)	Self-monitoring of behavior, instruction on how to perform the behavior, demonstration of the behavior, habit formation, nonspecific reward, self-reward, reduce negative emotions, and focus on past success

^a^TTM: transtheoretical model [32,33].

In addition to the theoretical model of behavior change, behavior change techniques (BCTs) are used. BCTs are active components that aim to change behaviors as a part of an intervention [38,39]. Here, the appropriate BCTs are selected in relation to the individual phases of the TTM (Tables 1 and 2).

**Figure 1:**
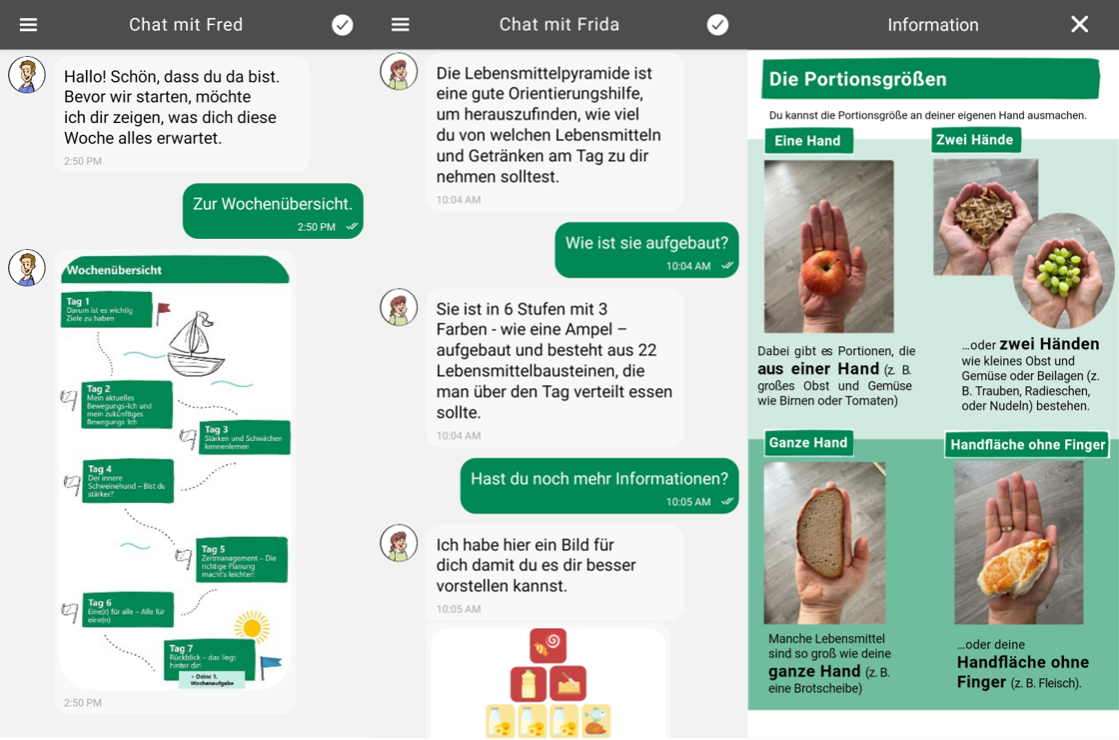
Screenshots of the mobile app. The information in the app is provided as text messages, graphics, and videos or by gamification and storytelling approaches. Screenshots taken on EMUI (version 12.0.0, Huawei).

The DHI is implemented in a mobile app using the MobileCoach intervention platform [40,41], an open-source platform for the design and deployment of DHIs based on rule-based CA. Here, the participant chooses 1 of 4 coaches before the intervention, and the intervention information is provided as text messages, graphics, and videos or by gamification and storytelling approaches by emulating human-like interactions (Figure 1). The participants receive new intervention content once a day (at midnight). Additional content is sent only after the previous day’s content is completed. Push notifications are sent once a day to motivate the participants to complete the intervention content.

The intervention content and mobile app were evaluated in a pretest [42]. In the pretest, the long-term unemployed participants (N=12) were asked to test the mobile app for 9 weeks, and feedback was recorded every 3 weeks. The intervention content and mobile app were modified as suggested by the participants.

### Randomized Controlled Intervention Study

#### Study Design

To elucidate the effectiveness of the designed intervention, a 2-armed parallel-designed RCT is conducted with the long-term unemployed of the rural areas of Germany. The study protocol has been approved by the ethics committee of the State Medical Chamber of Baden-Wuerttemberg, Germany (number F-2019-106), and registered in the German Clinical Trials Register (DRKS00024805).

The intervention group (n=100) receives information materials (eg, brochures) regarding healthy nutrition and physical activity behavior and has access to the DHI in the form of a mobile app (refer to the section Intervention Contents and Mobile App). The intervention period is set to be 9 weeks, as this has been shown to be in the range of adequate time spans for DHIs (usually between 4 and 12 weeks) [43-46]. At baseline, after 3 and 6 weeks of intervention, the participants in the intervention group are scheduled to visit the study center located in the affiliated social sector institution to attend additional face-to-face sessions regarding technical instructions on the mobile app, healthy nutrition, and physical activity behavior, respectively. The control group (n=100) will meet at baseline at the study center to receive information material in the form of, for example, brochures (same as the intervention group) but will not have access to the DHI and will not attend the face-to-face sessions regarding technical instructions, healthy nutrition, and physical activity behavior. In addition, all participants (intervention and control group) will visit the study center after 9 weeks of intervention for a joint conclusion and to receive an incentive of €50 (US $49.7). The group allocation will not be blinded, as this is not applicable to the study design. If possible, investigators analyzing the data will be unaware of the group assignment.

**Figure 2:**
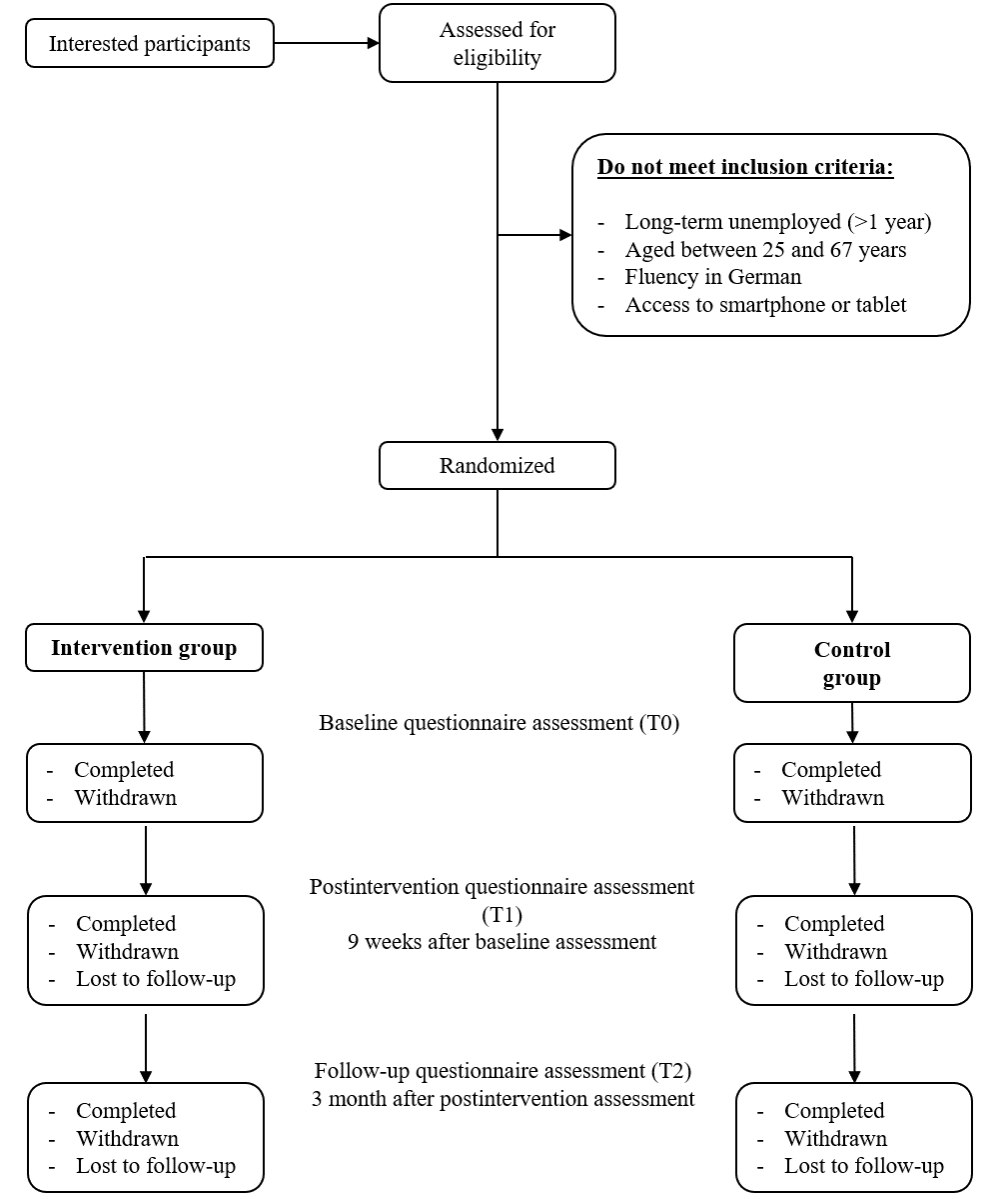
Participant flow diagram. Interested participants will be assessed for eligibility, and participants not meeting the inclusion criteria will be excluded. Remaining participants will be randomized into two groups (intervention and control). The completion, withdrawal, and losses to follow-up will be monitored, and the effectiveness of the intervention will be assessed at baseline (T0), after intervention (T1), and at follow-up (T2) by questionnaire assessment.

Recruitment is conducted via the social sector (eg, employment societies) of the rural regions in southwestern Germany, and participant recruitment started in March 2022. Recruitment will proceed until the required number of participants is reached or until the end of 2022. The inclusion criteria are long-term unemployment (defined as >12 months), between 18 and 67 years of age, and fluency in German. In addition, the participants must have access to a smartphone or tablet with Android (minimum version 6.0) or iPhone (minimum version 9.0) operating system. As the focus of this study is on rural areas, the affiliated social sector institutions must be located in rural areas. The long-term unemployed volunteers will be allocated randomly (Figure 2) to 1 of the 2 groups by a randomization list with a block size of 4, stratified to the social sector institutions, which accomplished the recruitment.

#### Outcomes

The effectiveness of the DHI will be evaluated by self-reported questionnaire assessment in German at baseline (T0), after 9 weeks of intervention (T1) at the study center, and after a 3-month follow-up period (T2) by mail services. Physical activity is assessed using the International Physical Activity Questionnaire in the German short version. The International Physical Activity Questionnaire has been validated several times [47,48] and measures the duration (minutes) and frequency (days) of sitting, moderate physical activity, and vigorous physical activity during the last 7 days, which will be expressed in minutes per day.

The food frequency questionnaire (FFQ) is a revised version of the FFQ used in the German Health Interview and Examination Survey for Adults (DEGS) [49], which was validated by the study by Haftenberger et al [50]. The FFQ consisted of 68 questions regarding the consumption frequency and portion sizes of food items in the past 4 weeks. The food intakes assessed by the FFQ will be expressed as servings of the consumed food item per day, and a food-based diet quality score will be analyzed according to the study by Masip et al [51].

To elucidate the adherence of the participants, the use of the mobile app will be monitored throughout the study period, and the affinity to technological devices is assessed by questionnaires before the study. The usability and acceptance of the mobile app will be assessed according to the unified theory of acceptance and use of the technology model [52]. Therefore, qualitative interviews or focus groups with the participants will be conducted and analyzed by structured content analysis according to the method by Mayring [53] using MAXQDA (version 2022; VERBI GmbH).

#### Statistical Analysis

Sample size calculation was conducted using G*Power (version 3.1.9.7, Heinrich Heine University Duesseldorf). As shown in a meta-analysis by Duan et al [54], the postintervention effect sizes of nutrition- and physical activity–related outcomes in patients with noncommunicable diseases in the intervention and control groups range widely from −1.11 to 6.40 (mean 0.85, SD 1.48) and −0.13 to 4.78 (mean 0.78, SD 1.77), respectively. As we suggested a lower adherence and thus a lower effect size in the targeted group in this study (long-term unemployed), we assumed the effect to be of medium size (Cohen *d*=0.5). With an alpha error of .05 and a power (1-β) of .80, a total sample size of 128 participants (n=64 per group) was calculated. In addition, a previously conducted pretest showed a high dropout of participants of approximately 33% after 9 weeks of treatment, defined as participants who did not reach the “action” phase according to Prochaska et al [33]. Thus, to take further potentially high dropout rates into account, we aim to include a total of 200 participants (n=100 per group) in this study.

To investigate the impacts of the intervention on the self-reported health outcomes assessed by the questionnaire, differences between the 3 data collection times (T0, T1, and T2) and between the 2 groups (intervention vs control) will be analyzed. To test for normal distribution, all data will be subjected to the Kolmogorov-Smirnov test. In case of normal distribution and homoscedasticity of variance (Mauchly’s sphericity test), time-dependent differences will be analyzed by repeated measurement analysis of variance with post hoc comparison by Bonferroni test. In case of nonparametric data, the Friedman test will be used, and the significance level will be corrected using Bonferroni correction. If a normal distribution is given, the differences between the 2 groups at 1 time point will be compared by 2-tailed *t* test. Otherwise, the data will be analyzed by Mann-Whitney *U* test. All differences will be considered statistically significant with *P* values <.05. Statistical analysis will be conducted using SPSS Statistics (version 28, IBM Corporation).

### Ethics Approval

This study was approved by the ethics committee of the State Medical Chamber of Baden-Wuerttemberg, Germany (number F-2019-106), and registered in the German Clinical Trials Register (DRKS00024805; registered on February 22, 2022). Written consent is obtained from all the participants before enrollment in this study.

## Results

The anticipated data will show whether the blended intervention can improve the nutritional or physical activity behavior of long-term unemployed in the rural areas of Germany. Individual effects of the intervention period and differences between the intervention and control groups will be elucidated. Furthermore, data on the use of the mobile app will show the adherence of the target group. In addition, the usability and acceptance of the mobile app will be evaluated.

Study enrollment started in March 2022. Study completion is due at the end of 2022. The first study outcomes are expected to be available in the spring of 2023. The data will be published in international peer-reviewed journals.

The study is part of the project “eHealth solutions to promote dietary and physical activity behaviors among the long-term unemployed in rural areas,” which is funded by the Federal Ministry of Education and Research (BMBF) since 2019 until 2023 (after a COVID-19 pandemic–related extension).

## Discussion

### Principal Findings

In this study protocol, a customized DHI to promote the nutritional and physical activity behaviors of long-term unemployed is presented. The mobile app will be tested in an RCT with long-term unemployed volunteers in a parallel-armed design in 2022.

### Comparison With Prior Work

Improving nutrition literacy and promoting physical activity are suggested to be successful strategies for improving the health of long-term unemployed. However, health interventions for the unemployed are scarce [55]. Moreover, they often fail to improve physical health [56,57], suggesting that either (1) the intervention design of these studies was inappropriate or (2) the intervention medium was not suitable for the targeted group. Regarding the intervention design, the intervention concept in this study is based on the TTM. The TTM is a stage-based behavior change model, which was first described for the cessation of smoking [33] and was proven to be effective by a review of Spencer et al [58]. The TTM has also been successfully used in behavior change interventions regarding nutritional (reviewed in the study by Nakabayashi et al [59]) and physical activity behaviors (reviewed in the study by Adams and White [60]), suggesting that the TTM is an effective model to change the nutritional and physical activity behaviors of long-term unemployed. However, the effectiveness in changing the behaviors is also discussed controversially [61-63], especially in regard to long-term behavior changes [60].

The intervention was conceived in the form of a DHI, which has been proven earlier to be a successful intervention medium for promoting healthier eating [25] and physical activity choices [26] in vulnerable groups. However, to date, there is no DHI specifically designed to meet the needs of long-term unemployed. Although it has been shown that mobile phone ownership is not considered a serious barrier to participate in DHIs, even for unemployed individuals [64], the accessibility to a DHI, and therefore its effectiveness, can be improved by considering support for older devices, the possibility of offline use, low digital literacy requirements, and the involvement of the targeted people in the development process [65]. Thus, the current intervention addresses health-related topics identified by the participative input of the long-term unemployed in Germany through interviews and workshops [31]. The conceived intervention is then realized by low-threshold informational content, supported by graphical files, links, and videos. A key point of the DHI is the use of a rule-based CA, which emulates an informal human-like interaction and is suggested to be encouraging and motivating [24]. Another strength of the present intervention is the blended use of an easily accessible DHI in the form of a mobile app in combination with classical face-to-face appointments. Face-to-face interventions have been proven to be more effective than interventions designed solely in a digital format [66-68]. However, DHIs are easily accessible to a wide range of the population and are associated with lower financial and time commitments, as they can be used at any time at home. Blended interventions, the combination of DHIs with face-to-face interventions, have been used frequently for the treatment of mental disorders in adults (reviewed in the study by Erbe et al [69]) and are discussed to be feasible and more effective than stand-alone interventions in the treatment of substance abuses, as they significantly reduce the participant dropout rates [70-73]. In the planned study, the conception of the intervention in a blended design is expected to decrease the participant dropout and, therefore, increase the intervention effectiveness. To minimize an increased effort to participate in face-to-face appointments, they will be located in the social sector societies near to the long-term unemployed in the rural areas of Germany, where they have, in general, daily face-to-face appointments with the employees of the social societies. However, the recruitment of social societies that are willing to assist the study by providing long-term unemployed participants and the infrastructure to carry out face-to-face appointments might be difficult, especially under the complicated circumstances due to the COVID-19 pandemic (eg, hygiene and distance rules).

### Limitations

This study has some limitations. First, we did not adapt the intervention according to the multiphase optimization strategy, which maximizes the translation of research into effective practice [74]. However, we did run a pretest while developing the intervention to ensure the feasibility of the blended intervention. Second, the effectiveness of the intervention is based on questionnaires and, therefore, might be underlying a reporting bias. Further studies might be necessary to measure physiological adaptation to a healthier lifestyle, for example, by measures of health-related biomarkers, such as markers for metabolic diseases [75] or cardiovascular health [76]. Third, as the participants receive incentives, they might have been overly motivated to use the mobile app. To counteract a potential overestimation of the app use through the incentives, we instructed the participants that the incentives are only tied to the completion of the questionnaire and not to the use of the mobile app. Finally, although in previously conducted interviews (N=20), most of the long-term unemployed in the rural areas of southwestern Germany (75%) had smartphones and regularly used mobile apps on their devices (Mages-Torluoglu, J, unpublished data, February 2020), some may not and, therefore, cannot participate in this study. However, the ownership of smartphone devices is still rapidly growing [77]. Thus, we feel that a DHI with face-to-face appointments is a promising strategy to improve the health of long-term unemployed in the rural areas of Germany. At this point, it must also be emphasized that behavior change strategies may only address the secondary effects of long-term unemployment. In general, improving living conditions (eg, better financial support or social inclusion) may be a more comprehensive strategy for promoting health among the unemployed.

### Conclusions

The intervention presented in this study is tailored to the needs of the long-term unemployed and is implemented in a blended intervention consisting of a DHI based on the interaction with an internet-based CA and additional face-to-face interactions. The effectiveness of the blended intervention in promoting improved nutritional and physical activity behaviors of long-term unemployed participants will be evaluated in a protocolized RCT and measured by questionnaire assessment. If we could show that the health intervention designed for the study described above is effective in promoting beneficial choices regarding nutrition (eg, eating more fruits and vegetables) and physical activity (eg, improving endurance), this would be a first and an important step in promoting the general health of the unemployed. The blended intervention is intended for implementation in community health systems and can thus contribute to an improved health status of the German population in general.

## Abbreviations

BCT: behavior change technique
CA: conversational agent
DHI: digital health intervention
FFQ: food frequency questionnaire
MI: myocardial infarction
RCT: randomized controlled trial
TTM: transtheoretical model

## Data Availability

The data sets generated or analyzed during this study are available from the corresponding author upon reasonable request.
